# Efficacy and Safety of Omalizumab for the Treatment of Severe or Poorly Controlled Allergic Diseases in Children: A Systematic Review and Meta-Analysis

**DOI:** 10.3389/fped.2022.851177

**Published:** 2022-03-15

**Authors:** Ling Liu, Pengxiang Zhou, Zhenhuan Wang, Suodi Zhai, Wei Zhou

**Affiliations:** ^1^Department of Pediatrics, Peking University Third Hospital, Beijing, China; ^2^Department of Pharmacy, Peking University Third Hospital, Beijing, China; ^3^Institute for Drug Evaluation, Peking University Health Science Center, Beijing, China; ^4^Department of Pharmacy, First Hospital of Tsinghua University, Beijing, China

**Keywords:** omalizumab, severe allergic disease, children, systematic review, meta-analysis

## Abstract

**Objective:**

To evaluate the efficacy and safety of omalizumab in the treatment of severe or uncontrolled allergic diseases in children.

**Methods:**

We conducted a systematic search of the PubMed, Embase, CENTRAL, and clinicaltrials.gov databases up to 23rd July 2021, with no language limitations. Randomised controlled trials (RCTs) comparing omalizumab with other treatments or placebo in children with severe or inadequately controlled allergic diseases were considered. The primary outcomes of interest were asthma exacerbation rate, allergic symptom score, desensitisation achievement for food allergy (FA), and incidence of serious adverse events (SAEs). The study selection and data extraction were conducted independently by two researchers. Quality assessments were conducted using the Cochrane risk-of-bias tool, and data were pooled using a random-effects model if *I*^2^ was 50% or greater in the Cochrane Review Manager.

**Results:**

Overall, 10 RCTs [six on severe asthma, one on atopic dermatitis (AD), one on seasonal allergic rhinitis [SAR], and one on FA] consisting of 2,376 participants met the inclusion criteria. For severe asthma, omalizumab may reduce exacerbations at 12 weeks [risk ratio (RR), 0.52; 95% confidence interval (CI), 0.31–0.89], 24 weeks (RR, 0.69; 95% CI, 0.55–0.85; GRADE: moderate-quality evidence), and 52 weeks (RR, 0.62; 95% CI, 0.40–0.94; GRADE: moderate-quality evidence) and reduce the dose of inhalation corticosteroid compared with placebo. For severe AD, the association between omalizumab and allergic symptom improvement [i.e., SCORing Atopic Dermatitis or Paediatric Allergic Disease Quality of Life Questionnaire (PADQLQ)] was not confirmed. For severe SAR, omalizumab showed greater improvement in symptom load scores and saved rescue medication days. For FA, omalizumab demonstrated superiority in desensitisation compared with placebo. To date, no clinically significant drug-related SAEs have been reported.

**Conclusion:**

For severe or uncontrolled asthma, AD, SAR, and FA, omalizumab may be associated with improved allergic symptoms and safety in children. Future studies should focus on the benefits and pharmacoeconomic evaluation of omalizumab in multiple allergic diseases compared with other treatments.

**Systematic Review Registration:**

[https://www.crd.york.ac.uk/PROSPERO], identifier [CRD42021271863].

## Introduction

Severe allergic diseases, which are not uncommon in children, can have a serious impact on children’s health, leading to death and heavy social and economic burdens (1). Approximately 5–10% of children worldwide are diagnosed with severe asthma (2), while approximately 20% of Chinese children suffer from uncontrolled asthma (3). Severe or poorly controlled asthma may cause decreased lung function, an increased risk of asthma exacerbation, higher hospitalisation rates, increased unplanned hospital visits, and increased medical costs. In addition, other severe allergic diseases can result in adverse conditions. For instance, food allergies (FAs) may cause anaphylaxis or even death. Patients with severe atopic dermatitis (AD) may experience sleep disruption, resulting in significantly increased morbidity, poor school performance, and psychiatric disorders (4). Children with moderate-to-severe allergic rhinitis (AR) often have recurrent uncontrolled asthma and increased medical costs, likely leading to poor asthma control and chronic sinusitis (5). Furthermore, multiple allergic diseases might interact with each other and are closely associated with the severity of the condition. This phenomenon is more commonly observed in children (6, 7).

To date, there is no universally accepted and effective treatment for severe or uncontrolled allergic diseases in children, except for biologics, which can reduce inflammatory urticaria motion by blocking immunoglobulin E (IgE), interleukin (IL)-4, 5, 6, 17, and other inflammatory factors (8, 9). Among these, omalizumab is widely recommended in guidelines (10–12) and has been approved for the treatment of moderate-to-severe allergic asthma and chronic spontaneous urticaria. Omalizumab is a monoclonal antibody that targets circulating free IgE and prevents its interaction with the high-affinity IgE receptor, thereby interrupting the allergic cascade (13). Therefore, omalizumab may be a promising drug for treating children with severe or uncontrolled allergic diseases, even multiple allergic diseases.

To the best of our knowledge, there has been no comprehensive evaluation demonstrating the benefits and risks of omalizumab application in severe or uncontrolled paediatric allergic diseases. We conducted a systematic review and meta-analysis of randomised controlled trials (RCTs) on the efficacy and safety of omalizumab in children with severe allergies.

## Methods

This systematic review was conducted by referring to the Cochrane Collaboration and reported according to Preferred Reporting Items for Systematic Reviews and Meta-Analyses (PRISMA) Statement (14), with its protocol registered with PROSPERO (CRD42021271863).

### Search Strategy

We searched PubMed, Embase, the Cochrane Central Register of Controlled Trials (CENTRAL), and ClinicalTrials.gov databases from inception to 23rd July 2021 using medical subject heading terms and Emtree headings, mainly including omalizumab with children or paediatric population filters (Supplementary Table 1). There were no restrictions on the language or publication years. We also manually searched ClinicalTrials.gov for registered ongoing or recently completed trials, as well as reference lists of related reviews.

### Inclusion Criteria

We included RCTs that compared any dosage form of omalizumab with other treatments in children with severe or poorly controlled allergic diseases. We excluded animal experimental trials and publications that did not undergo peer review, such as letters, editorials, and opinions. Two reviewers (LL and PZ) independently screened the titles, abstracts, and full-text reports for eligibility, with disagreements resolved by discussion or intervention by a third coordinator (ZW). The authors were contacted if there was insufficient data of the included records, when necessary.

### Data Extraction and Analysis

Two reviewers (LL and PZ) independently extracted the basic information and outcomes using a pre-specified form. The basic information included the first author, publication year, participants, countries, single-or multi-centre conditions, cases, ages, genders, treatment durations, interventions, comparisons, efficacy outcomes, and safety outcomes.

For different allergic diseases, the primary outcome measures were as follows: the incidence of exacerbations for allergic asthma; symptom load score (eyes and nose) for AR; SCORing Atopic Dermatitis (SCORAD) index for AD; and desensitisation achievement for FA. For safety, the rates of serious adverse events (SAEs) or adverse events (AEs) were identified as the primary outcomes. Additional outcome measures were the dosage of inhaled corticosteroid or rescue medication needed to maintain asthma control, Childhood Asthma-Control Test (C-ACT) improvement, and lung function FEV1 for allergic asthma; usage of rescue medication for AR; the incidence of passing the double-blind placebo-controlled food challenge (DBPCFC) for FA; and the quality-of-life Paediatric Allergic Disease Quality of Life Questionnaire (PADQLQ) for AD.

### Quality of Evidence

Two reviewers (PZ and ZW) independently evaluated the risk of bias for each RCT using the Cochrane risk-of-bias tool (15). Overall quality of evidence for each outcome was assessed using the Grading of Recommendations Assessment, Development and Evaluation (GRADE) approach (16). Any disagreements in the evaluation were resolved by discussion or by a third reviewer (SZ).

### Statistical Analysis

The intervention effects were estimated by calculating the risk ratio (RR) or mean difference (MD) with 95% confidence intervals (CIs). Meta-analyses were performed for continuous and categorical outcomes using Review Manager (RevMan) version 5.4.0. Heterogeneity among studies was calculated using the *I*^2^ statistic in RevMan. The fixed-effects model was used when *I*^2^ was less than 50%; otherwise, a random-effects model was chosen. A two-tailed *P*-value of <0.05 was considered statistically significant. If there were insufficient data for outcome synthesis, narrative analyses were conducted.

Subgroup analyses were systematically performed according to different allergic diseases, controls, and allergens, when possible. Publication bias was assessed using funnel plots of the main outcomes. Sensitivity analyses were performed by excluding trials with a high risk of bias.

## Results

### Study Characteristics and Risk of Bias Assessment

**FIGURE 1:**
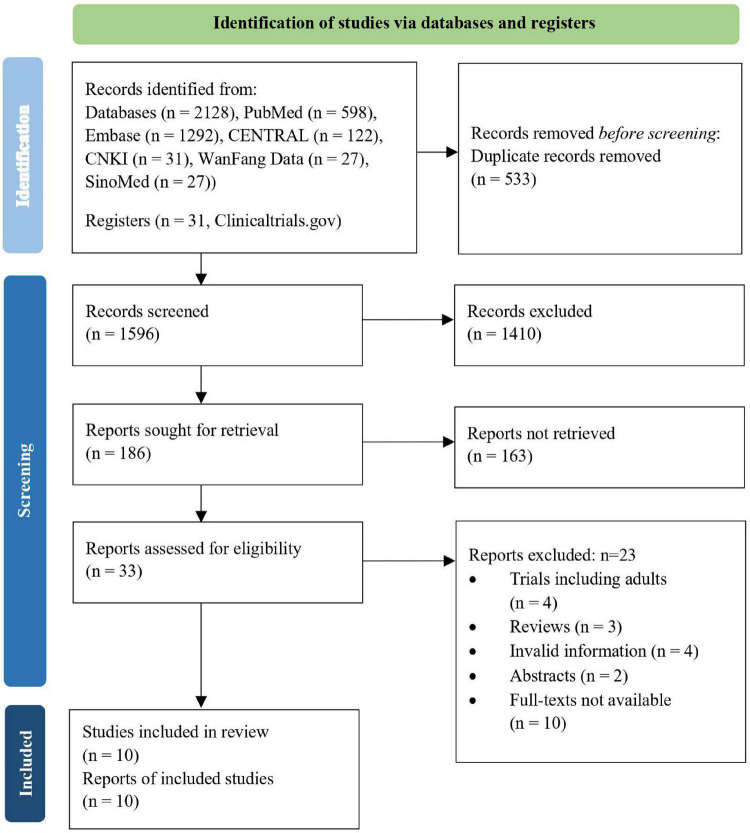
Flow diagram of study selection process.

Overall, we retrieved 2,128 references and assessed 33 full-text articles for eligibility. Ten RCTs involving 2,376 participants were finally selected for qualitative or quantitative analyses (Figure 1).

**FIGURE 2:**
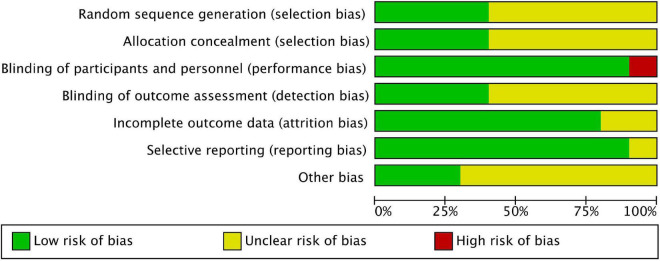
Risk of bias graph.

**FIGURE 3:**
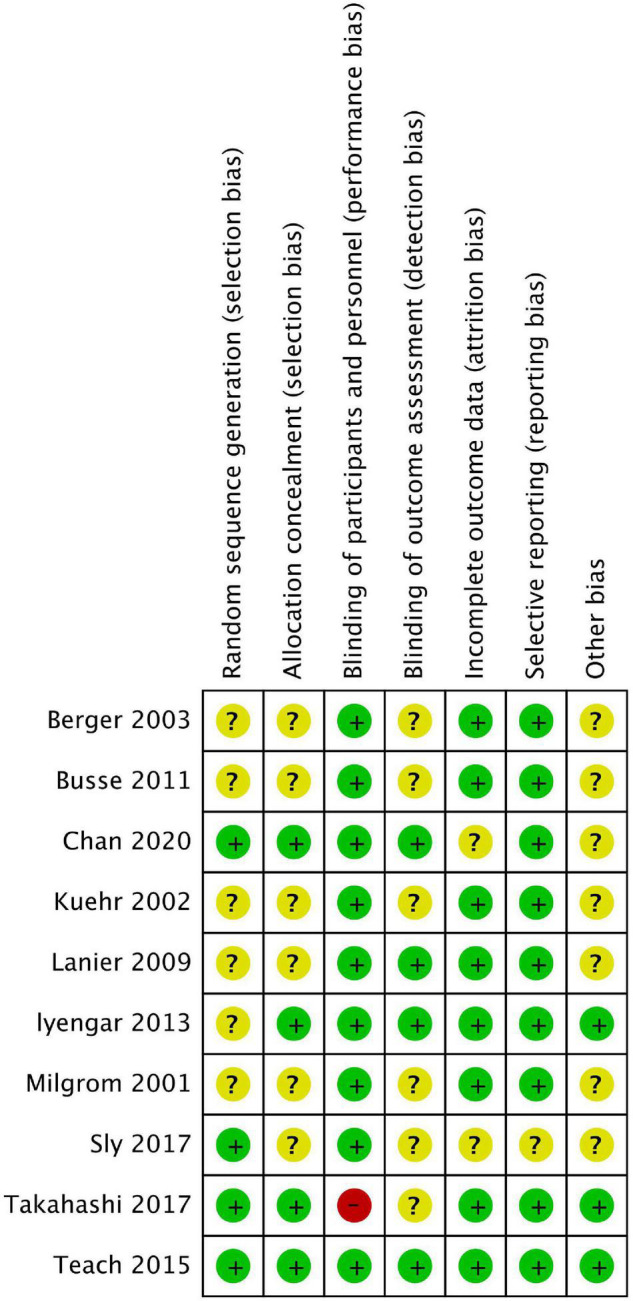
Risk of bias summary.

Among these studies, six were related to severe allergic asthma, two to AD, one to seasonal AR (SAR), and one to FA. Table 1 shows the basic characteristics of the included studies. The results of the risk of bias are summarised in Figures 2, 3, with the detailed reasons in Supplementary Table 2. Except for one open-label trial (17), the majority of included trials were evaluated as having a low risk of performance bias.

**TABLE 1:** Basic characteristics of included RCTs.

References	Participants	Countries-centre	Cases	Age	Gender	Treatment duration	Usage and dosage in intervention group and control group	Efficacy outcome measures	Safety outcome measures
							
			(T/C)	(T/C)	(T/C, female %)				
Berger et al. (18)	Moderate-to-severe allergic asthma	United States; multi-centre	225/109	9.4 (5–12)/NA	29.8%/NA	28 weeks (core study) and 24 weeks (open-label)	Active group: OMB 150 or 300 mg every 4 weeks or 225, 300, or 375 mg every 2 weeks; Control group: placebo	Main outcomes: BDP use; asthma exacerbations. Secondary outcomes: pulmonary function assessments; rescue asthma medication.	AE; SAE
Milgrom et al. (24)	Moderate-to-severe allergic asthma	United States; multi-centre	225/109	9.4 (5–12)/9.5 (6–12)	29.8/33%	28 weeks (core study) and 24 weeks (open-label)	Active group: OMB 150 or 300 mg every 4 weeks or 225, 300, or 375 mg every 2 weeks; Control group: placebo	Main outcomes: BDP use; asthma exacerbations. Secondary outcomes: pulmonary function assessments; rescue asthma medication.	AE; SAE
Busse et al. (21)	Persistent asthma	United States; multi-centre	208/211	10.9 ± 3.6/ 10.8 ± 3.4	41/43%	60 weeks	Active group: OMB 75–375 mg, ≥0.016 mg/kg/IU/mL Control group: placebo	Main outcomes: exacerbations; the dose of ICS needed to maintain asthma control; C-ACT. Secondary outcomes: FEV1.	AE; SAE
Lanier et al. (19)	Persistent allergic asthma	International; multi-centre	384/192	8.7 ± 1.7/ 8.4 ± 1.7	31.8/33.3%	52 weeks (24-week fixed-steroid phase and a 28-week adjustable-steroid phase)	Active group: OMB 75–375 mg, every 2 or 4 weeks; Control group: placebo	Main outcomes: asthma exacerbations; C-ACT; percentage reduction in ICS dose. Secondary outcomes: rescue medication use.	AE
Teach et al. (22)	Persistent asthma	United States; multi-centre	259/130/89	10.3 ± 2.99/ 9.84 ± 2.7/ 10.1 ± 3.06	33.8/46.2/33.7%	90 days	Active group: OMB based on weight and serum IgE levels, every 2 or 4 weeks (75–375 mg); Control group 1: ICS boost: doubled the ICS dose Control group2: placebo	Main outcomes: asthma exacerbations.	AE; SAE
Sly et al. (23)	Persistent asthma	Australia; multi-centre	14/13	11.51 ± 2.94/ 11.4 ± 3.15	46/54%	5 months	Active group: OMB based on total IgE level, every 2–4 weeks (75–375 mg); Control group: placebo	Main outcomes: asthma exacerbations.	NA
Chan et al. (25)	Severe eczema	United Kingdom; single-centre	30/32	10.2 ± 0.1/ 10.4 ± 4.3	57/41%	24 weeks of treatment with an additional 24 weeks of follow-up	Active group: OMB based on weight and total IgE level (75–37 5mg); Control group: placebo	Main outcomes: total SCORAD. Secondary outcomes: PADQLQ.	AE; SAE
Iyengar et al. (26)	Severe refractory AD	United States; single-centre	4/4	7.4/15.8	NA	24 weeks	Active group: OMB 150–375 mg, every 2 or 4 weeks. Control group: placebo	Main outcomes: SCORAD.	NA
Kuehr et al. (27)	SAR	Germany; multi-centre	54/55/53/59	12 (6–17)	42.6: 43.6: 28.3: 52.5%	24 weeks	Active group: OMB based on weight and total IgE level, ≥0.016 mg/kg/IU/mL of IgE, every 4 weeks; Control group: placebo	Main outcomes: the symptom load; rescue medication score.	AE; SAE
Takahashi et al. (17)	Persistent cow’s milk allergy	Japan; single-centre	10/6	9.5/9.5	50%/0	24 weeks	Active group: OMB-OIT, OMB based on weight and total IgE level every 2 or 4 weeks (75–375 mg); Control group: placebo	Main outcomes: the incidence of passing DBPCFC by the OMB-OIT treatment.	AE; SAE

*T, Trial groups; C, control groups; OMB, omalizumab; AD, atopic dermatitis; SAR, seasonal allergic rhinitis; BDP, beclomethasone dipropionate; ICSs, inhaled corticosteroids; SCORAD, SCORing Atopic Dermatitis; PADQLQ, Paediatric Allergic Disease Quality of Life Questionnaire; C-ACT, the score on the Childhood Asthma Control Test; OIT, immuno-therapy treatment; DBPCFC, double-blind placebo-controlled food challenge.*

### Severe Asthma

#### Exacerbations

**FIGURE 4:**
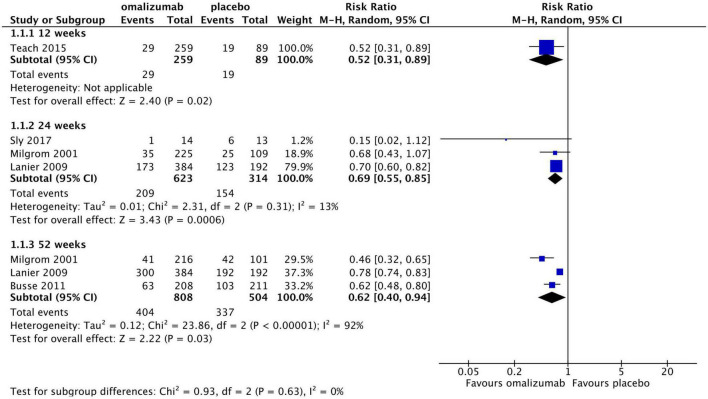
Comparison of omalizumab with placebo for severe asthma exacerbations.

For categorical asthma exacerbation rate, moderate-quality evidence without high risk of bias showed that compared with placebo, omalizumab was significantly associated with a lower rate of asthma exacerbations over a period of 12 weeks (one study with 348 patients; RR, 0.52; 95% CI, 0.31–0.89; *P* = 0.02), 24 weeks (three studies with 937 patients; RR, 0.69; 95% CI, 0.55–0.85; *P* < 0.0006, *I*^2^ = 13%; GRADE: moderate quality evidence, with serious bias in imprecision), and 52 weeks (three studies with 1,312 patients; RR, 0.62; 95% CI, 0.40–0.94; *P* = 0.03, *I*^2^ = 92%; GRADE: moderate quality evidence, with serious bias in inconsistency) (Figure 4; GRADE: Supplementary Table 3). Considering the potential clinical or methodological heterogeneity among trials, sensitivity analyses were conducted by excluding each included trial, and a random model was chosen to synthesise the data (18–24).

#### Inhalation Corticosteroid or Rescue Treatment

The lower the inhalation corticosteroid (ICS) used, the better the treatment effect. From baseline to 28 weeks, the median dose of beclomethasone dipropionate (BDP) was reduced by 100% in the omalizumab group compared with that in the placebo group (66.7%, *P* = 0.001) (18). At 28 weeks, BDP was not used again in 55% of the patients in the omalizumab group compared to 39% in the control group (*P* = 0.004). Patients treated with omalizumab had a significant reduction in the dose of ICS compared with that in the placebo group (109 μg less per day, 95% CI, −172 to −45; *P* < 0.001) (21). After omalizumab treatment, the ICS dose was slightly decreased by 4%, whereas there was a 2% increase in ICS dose in the placebo group (19). The less concomitant medication use in severe asthma indicated a better asthma control. Daily rescue inhaled medication in the omalizumab group was less than that in the placebo group (−1.3 puffs ±2.84; −1.0 puffs ±2.50, *P* < 0.047) (19).

#### Childhood Asthma-Control Test Score

Higher C-ACT scores reflect better asthma control. Busse et al. (21) showed that after treatment for 48 weeks, the C-ACT score was 0.78, higher in the omalizumab group than in the placebo group (95% CI, 0.21–1.35; *P* = 0.007) among children aged 4–11 years.

### Quality of Life

Only one trial showed quality of life in children with severe allergic asthma after using omalizumab, and there was no significant difference between the groups (19).

#### Pulmonary Function Assessments

FEV1 is one of the most common laboratory indices that reflects the level of asthma control. Only one trial demonstrated no significant difference in FEV1 between the omalizumab (92.6 ± 0.60) and placebo groups (91.7 ± 0.64) (MD = 0.92; 95% CI, −0.81 to 2.64; *P* = 0.30), and there was no significant difference in FEV1/FVC between the omalizumab (77.3 ± 0.36) and placebo groups (77.5 ± 0.38) (MD = −0.13; 95% CI, −1.16 to 0.91; *P* = 0.81) (21).

### Severe Atopic Dermatitis

#### SCORing Atopic Dermatitis and Paediatric Allergic Disease Quality of Life Questionnaire

The greater the decrease in the SCORAD score, the better the treatment effect on AD. Two studies have reported SCORAD scores following treatment (25, 26). Chan et al. (25) showed that omalizumab was clearly superior to placebo (MD = −6.9; 95% CI, −12.2 to −1.5; *P* = 0.01), while Iyengar et al. (26) demonstrated that there was no significant difference between the two groups among eight patients with severe refractory eczema after 24 weeks (20–50% decrease in the omalizumab group; 45–80% in the placebo group). Only one study (25) reported PADQLQ changes in children with severe AD, and the results showed no difference in PADQLQ between the omalizumab and placebo groups (MD = 0.04; 95% CI, −0.9 to 1.0; *P* = 0.933).

### Severe Seasonal Allergic Rhinitis

#### Symptom Load Score

Kuehr et al. (27) reported that the symptom load score was 48% lower in the specific immunotherapy treatment (SIT) combined with omalizumab group than in the SIT alone group (*P* < 0.001). In the subgroup analysis, the combination group had a 71% reduction in symptom load score compared to that in the placebo group during the grass pollen season (*P* < 0.001).

#### Rescue Medication Usage Days

The fewer the days of rescue medication use, the better the treatment effect. Days of rescue medication was significantly less in patients treated with a combination of omalizumab and SIT than in those receiving placebo during the entire pollen season (median percent of days: 4.36 vs. 15.07; *P* < 0.001) (25).

### Food Allergy

#### Persistent Cow’s Milk Allergy

The higher the tolerance to cow milk or the more DBPCFC passed, the better cow milk allergy was controlled. Takahashi et al. (17) reported that all patients treated with a combination of omalizumab and oral immunotherapy (OIT) achieved desensitisation, while none of the patients in the placebo group achieved desensitisation.

#### Safety

Two studies showed that the incidence of AEs was similar between the omalizumab and placebo groups (47.4 vs. 39.4%, *P* = 0.06; 54.5 vs. 54.8%, *P* > 0.99) (21, 22). Lanier et al. (19) reported that the incidence of AEs in the omalizumab group was lower than that in the control group (90 vs. 93.7%, *P* < 0.05), whereas Berger et al. (18) arrived at the opposite conclusion (89.3 vs. 87.2%, *P* < 0.05). Although the incidence of SAEs was 3.6–13.7%, only three cases were judged to be related to omalizumab, including one case of generalised urticaria (18–20, 25), one case of moderate tic disorder (19), and one case of anaphylaxis 10 h after the third injection (25). Detailed information on the time of occurrence, manifestations, and prognosis of SAEs has not been reported, and there were no drug-related death events.

## Discussion

This systematic review based on moderate-to-low quality evidence demonstrated that omalizumab showed satisfactory efficacy and tolerance in the control of severe or uncontrolled allergic asthma, severe AD, and SAR in children. Limited evidence has shown that omalizumab helps children with milk allergies achieve desensitisation. Our findings are partly supported by similar results from several previous systematic reviews on asthma (28–31), while adding up-to-date evidence to all types of severe allergic diseases in children, which fills the gap in this important issue.

Although several prior studies support the effect of omalizumab in the treatment of allergic diseases, the current indications for children are limited to moderate-to-severe allergic asthma and chronic spontaneous urticaria. However, other allergic children, such as those with AR and severe AD, have not been adequately treated with this drug because of the indication limits. This review would provide support for the expansion of omalizumab indications for allergic diseases in children and development of a new approach for children with multiple allergic diseases.

Although the current advantages of omalizumab are supported by limited controlled trials and clinical practice, the cost-effectiveness and risk of long-term use in children should be further investigated. In addition, considering that omalizumab is used in hospital settings and is time-consuming and inconvenient compared to other oral or inhaled treatments, paediatricians should comprehensively evaluate the severity of allergic diseases and provide appropriate recommendations.

To the best of our knowledge, there is no unified definition of severe allergic disease; however, there is a clear classification for the severity of severe allergic asthma (32), moderate-to-severe AR (33), and severe AD (34). Severe allergic diseases can cause both physical suffering and place economic burden on society. Moreover, it is worth mentioning that patients with severe allergic asthma sometimes have comorbidities, such as seasonal rhinitis, conjunctivitis, AD, and FA (35). The presence of these comorbid allergic diseases is not conducive to the improvement of the primary allergic diseases. Therefore, treatment of children with severe comorbid allergic diseases or non-responses to conventional anti-allergic treatments should be considered. However, there is currently no effective treatment for these children.

Children with severe allergic diseases require long periods of combined medications, including antihistamines, airway dilators, or oral, inhaled, and topical corticosteroids, to relieve symptoms. There is increasing recognition that omalizumab may have the potential to reduce combined medications and reduce the risk of side effects to some extent. However, other biologics, such as dupilumab, mepolizumab, reslizumab, benralizumab, have only been indicated for adolescents or adults, and thus omalizumab is the only biologic regimen indicated for children younger than 12 years old.

Patients with severe allergic diseases often exhibit high levels of Th2 inflammatory cytokines. They are mainly caused by type I allergic reactions that are mediated by IgE (36). Previously, a study reported that allergic asthma and other allergic comorbidities share the same underlying IgE-mediated pathophysiological mechanism (37). The immune mechanisms of AD are complex (38). IgE-mediated immune responses play an important role in severe AD in children compared to that in adults (39). As the pathogenesis is mostly related to IgE mediation, anti-IgE therapy may be a promising treatment for these children. Omalizumab is a monoclonal anti-IgE antibody that prevents free IgE from interacting with high-affinity IgE receptors on mast cells, basophils, macrophages, dendritic cells, and other cell types (40). Therefore, omalizumab may have good efficacy and acceptable safety for different categories of severe allergies and multiple allergic disorders in children.

Wood et al. (41) published the first RCT study of omalizumab combined with OIT for the treatment of cow milk allergy, and the results showed that omalizumab effectively improved the safety of desensitisation. However, this did not reflect the effect of treatment on desensitisation to cow milk allergy. The study included children and adults and did not focus only on children. By contrast, the study on milk protein desensitisation using liquid milk, which was part of Takahashi et al.’s study (17) on microwaved milk, is more suitable for assessing desensitisation. The authors reported that OIT tolerance can be improved by reducing allergic sensitisation through heating or other immunotherapeutic methods (42).

The key limitation of this review—already alluded to— is the small sample size of paediatric participants, and we only conducted meta-analyses of asthma exacerbations. A major clinical heterogeneity may result from the wide range of omalizumab dosages and different degrees of asthma progression. Therefore, we consequently downgraded the GRADE evaluation. In addition, this review only included participants under 18 years of age; therefore, trials recruiting patients aged 12–18 years and those older than 18 years were not considered. Moreover, we failed to further divide the limited patients into two cohorts according to the age range. Consequently, the efficacy and safety of omalizumab in adolescents are less strongly demonstrated in this review. Unfortunately, we could not find a trial indicating the use of omalizumab in chronic urticaria, thereby limiting its application in this allergic disease.

## Conclusion

This review suggests that omalizumab may be a promising treatment with satisfactory efficacy and safety in children with severe asthma, AD, SAR, or FA. Future studies should focus on the benefits and pharmacoeconomic evaluation of omalizumab in multiple allergic diseases compared with other treatments.

## Data Availability Statement

The original contributions presented in the study are included in the article/Supplementary Material, further inquiries can be directed to the corresponding author.

## Author Contributions

LL and PZ conducted the study registration, literature search, selection, review, and data analyses. ZW and PZ conducted quality assessment and GRADE evaluation. WZ and SZ provided the paediatric and pharmacological guidance. All authors participated in the research design, contributed to the writing of this manuscript, and approved the final version.

## Conflict of Interest

The authors declare that the research was conducted in the absence of any commercial or financial relationships that could be construed as a potential conflict of interest.

## Publisher’s Note

All claims expressed in this article are solely those of the authors and do not necessarily represent those of their affiliated organizations, or those of the publisher, the editors and the reviewers. Any product that may be evaluated in this article, or claim that may be made by its manufacturer, is not guaranteed or endorsed by the publisher.
