# Supplementary material for: Efficacy and Safety of Omalizumab for the Treatment of Severe or Poorly Controlled Allergic Diseases in Children: A Systematic Review and Meta-Analysis
Source: Front Pediatr. 2022 Mar 15;10:851177. doi: 10.3389/fped.2022.851177 (PMC8965060; doi:10.3389/fped.2022.851177)
Supplement: Supplementary file 1 [file Data_Sheet_1.pdf]

Supplementary Materials

Table S1 Search Strategies

Databases	Detailed search strategies
PubMed	("omalizumab"[MeSH Terms] OR "omalizumab"[All Fields] OR "omalizumab s"[All Fields]) AND (allchild[Filter])
Embase	('omalizumab'/exp OR omalizumab) AND ([newborn]/lim OR [infant]/lim OR [child]/lim OR [preschool]/lim OR [school]/lim OR [adolescent]/lim)
The Cochrane Library (CENTRAL)	(omalizumab):ti,ab,kw and (MeSH descriptor: [Child] explode all trees or MeSH descriptor: [Infant] explode all trees or MeSH descriptor: [Adolescent] explode all trees)
Clinicaltrials.gov	Omalizumab (with results)

Table S2 Reasons of Risk of Bias Assessment of Included Trials

Studies	Selection bias (Randomization)	Selection bias (Allocation concealment)	Performance bias	Detection bias	Attrition bias	Reporting bias	Other bias
Sly 2017	Randomization occurred in blocks stratified by study site based on predetermined block sizes	NA	Double-blind	NA	Only 27 from initial 96 participants were reported without declaring the reason	Safety data were not reported	NA
Teach 2015	Computer-based random allocation scheme	Centralized	Quadruple blind (participant, care provider, investigator, outcomes assessor)	Quadruple blind (participant, care provider, investigator, outcomes assessor)	Missing data did not affect the consistency between groups	All predesigned outcomes were reported	None
Lanier 2009	NA	NA	Quadruple blind (participant, care provider,	Quadruple blind (participant, care provider,	Missing data did not affect the consistency between groups	All predesigned outcomes were reported	NA

			investigator, outcomes assessor)	investigator, outcomes assessor)			
Takahashi 2017	Simple computerized randomization	Allocation was by sequentially numbered, opaque sealed envelopes by a hospital nurse	Open-label	NA	No missing data	All predesigned outcomes were reported	None
Kuehr 2002	NA	NA	Double blind	NA	Missing data did not affect the consistency between groups	All predesigned outcomes were reported	NA
lyengar 2013	NA	Central allocation	Triple blind (participant, care provider, investigator)	Triple blind (participant, care provider, investigator)	No missing data	All predesigned outcomes were reported	None
Chan 2020	Computer generated by the UK Clinical	Central allocation	Quadruple blind (participant, care provider,	Quadruple blind (participant, care provider,	One patient was withdrawn in the intervention	All predesigned outcomes were reported	NA

	Research Collaboration		investigator, outcomes assessor)	investigator, outcomes assessor)	group without reasons		
Busse 2011	NA	NA	Double blind	NA	Missing data did not affect the consistency between groups	All predesigned outcomes were reported	NA
Berger 2003	NA	NA	Double blind	NA	No missing data	All predesigned outcomes were reported	NA
Milgrom 2001	NA	NA	Double blind	NA	No missing data	All predesigned outcomes were reported	NA

Table S3 GRADE Evidence Profile

Certainty assessment						No of patients		Effect	Certainty
**No of studies**	**Risk of bias**	**Inconsistency**	**Indirectness**	**Imprecision**	**Publication bias**	**Omalizumab**	**Placebo**	**(95% CI)**	
Severe asthma exacerbations at 24 weeks									
3 (RCTs)	not serious	not serious	not serious	**serious**[a]	Undetected	623	314	RR 0.69 (0.55, 0.85)	⊕⊕⊕◯ moderate
Severe asthma exacerbations at 52 weeks									
3 (RCTs)	not serious	**serious**[b]	not serious	not serious	Undetected	808	504	RR 0.62 (0.40, 0.94)	⊕⊕⊕◯ moderate

CI: confidence interval; RR: risk ratio

GRADE Working Group grades of evidence

High certainty: We are very confident that the true effect lies close to that of the estimate of the effect.

Moderate certainty: We are moderately confident in the effect estimate; the true effect is likely to be close to the estimate of effect, but there is a possibility that it is substantially different.

Low certainty: Our confidence in the effect estimate is limited; the true effect may be substantially different from the estimate of the effect.

Very low certainty: We have very little confidence in the effect estimate; the true effect is likely to be substantially different from the estimate of effect.

a Downgraded once for imprecision due to wide confidence interval as a binary outcome.

b Downgraded once for inconsistency due to unexplained heterogeneity ($I^2 > 50\%$).
